# Strandings of cetaceans on the Espírito Santo coast, southeast Brazil, 1975–2015

**DOI:** 10.3897/zookeys.948.50468

**Published:** 2020-07-13

**Authors:** Luis Felipe S. P. Mayorga, Ralph E. T. Vanstreels, Renata C. C. Bhering, Natália Mamede, Luiz M. B. Costa, Flavia C. F. Pinheiro, Luciano W. D. Reis, Alessandro Trazzi, Wilson Luiz Chevitarese Meirelles, Alan Marques Ribeiro, Salvatore Siciliano

**Affiliations:** 1 Instituto de Pesquisa e Reabilitação de Animais Marinhos, Avenida Mário Gurgel S/N, Cariacica, Espírito Santo, Brazil; 2 Instituto Aqualie, Avenida Doutor Paulo Japiassú Coelho 714, Juiz de Fora, Minas Gerais, Brazil; 3 Alcom Indústria e Comércio, Rodovia BR-101 km 409, Itapemirim, Espírito Santo, Brazil; 4 Associação Ambiental Voz da Natureza, Rua Coronel Schwab Filho S/N, Vitória, Espírito Santo, Brazil; 5 Instituto Mamíferos Aquáticos, Rua dos Radioamadores 357, Salvador, Bahia, Brazil; 6 CTA–Servicos em Meio Ambiente, Avenida Saturnino Rangel Mauro 283, Vitória, Espirito Santo, Brazil; 7 A&R Consultoria e Treinamento, Rua Porfírio Furtado 125, Santa Leopoldina, Espírito Santo, Brazil; 8 Laboratório de Biodiversidade, Instituto Oswaldo Cruz/Fiocruz, Pavilhão Mourisco, sala 217, Av. Brasil, 4365, Manguinhos, Rio de Janeiro, RJ, 21040-900, Brazil; 9 Grupo de Estudos de Mamíferos Marinhos da Região dos Lagos, Rua São José 1260, Araruama, Rio de Janeiro, Brazil

**Keywords:** Atlantic Ocean, coast, estuary, Doce River, Odontoceti, Mysticeti, South America

## Abstract

Espírito Santo state is located on the eastern margin of Brazil, in a transitional tropical-subtropical area (18°S–21°S) dominated by oligotrophic waters. With the exception of humpback whales (*Megaptera novaeangliae*), the cetacean community of Espírito Santo has been understudied. In addition to the chronic impacts from fisheries, marine pollution, urban development, and coastal habitat degradation, in November 2015 the cetacean communities of Espírito Santo were challenged by the greatest environmental disaster in Brazil’s history. The Mariana dam disaster caused 60 million cubic meters of mining waste to be washed into the Doce River, which ultimately flowed to the coastal waters of Espírito Santo, with a high concentration of heavy metals. This study reviews and updates information on cetacean strandings in the state of Espírito Santo (excluding humpback whales) prior to this disaster. From 1975 to September 2015, there were 461 recorded cetacean strandings, representing 20 species. An average 1.18 strandings per 100 km per month were recorded since a state-wide daily beach survey program was implemented in October 2010, contrasting with the 0.14 strandings per 100 km per month in previous years. Six species comprised the majority (94.7%) of stranding events: Guiana dolphin (*Sotalia guianensis*), Franciscana (*Pontoporia blainvillei*), rough-toothed dolphin (*Steno bredanensis*), bottlenose dolphin (*Tursiops truncatus*), sperm whale (*Physeter macrocephalus*), and melon-headed whale (*Peponocephala electra*). Oceanic cetaceans stranded most frequently on the southern portion of Espírito Santo, where the continental platform is narrower, whereas the strandings of coastal cetaceans such as Guiana dolphins and Franciscanas were concentrated near estuaries, especially the Doce River. This is particularly concerning in face of the Mariana dam disaster, which drastically altered the estuarine and coastal environment associated with the Doce River.

## Introduction

Records of cetacean strandings provide reliable data on the occurrence of species and are good indicators of species richness, relative abundance and spatial distribution (Maldini et al. 2005, Pyenson 2010, 2011), and can be used to inform the management of marine resources (Leeney et al. 2008, Peltier et al. 2014). In coastal areas, cetaceans may be impacted by artisanal fisheries (de Freitas Netto and Di Beneditto 2007, 2008, de Freitas Netto and Siciliano 2007), marine traffic (e.g., acoustic pollution, collisions) (Pinheiro et al. 2019) and changes in geomorphology waves due to urban occupation of coastal areas (Albino et al. 2001, Ribeiro and Siqueira 2012).

**Figure 1.**
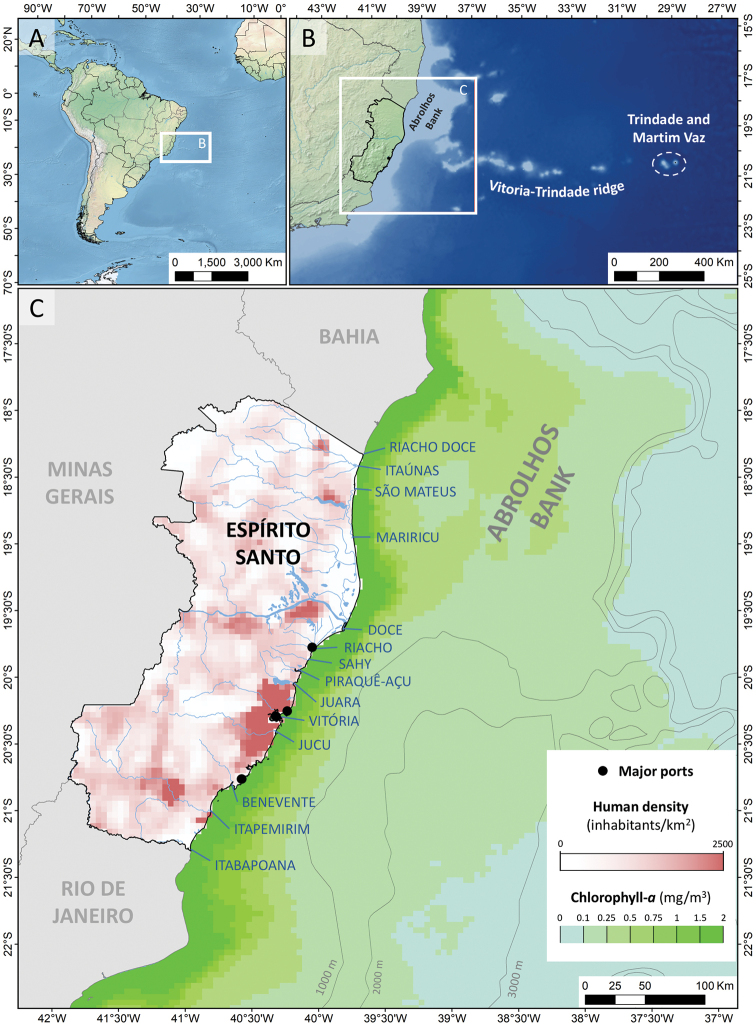
Physical, biological, and human characteristics of the coast of Espírito Santo state, southeast Brazil. Legend: **A, B** Location of Espírito Santo and Trindade and Martim Vaz Islands **C** human population density, major ports, isobaths, sea surface chlorophyll-*a* concentration, estuaries, and bays in the study area. Data sources: (Centro Internacional de Agricultura Tropical et al. 2005, Becker et al. 2009, NASA Earth Observatory 2019).

Espírito Santo state is located on the eastern margin of Brazil (Figure 1), in a transitional tropical-subtropical area (18°S–21°S). The marine environment of Espírito Santo hosts a substantial fish diversity (Floeter et al. 2007, Pinheiro et al. 2015a, b) and is an important winter breeding grounds of humpback whales (*Megaptera novaeangliae*) that migrate annually from the South Sandwich Archipelago (Siciliano 1995, Siciliano et al. 2012), constituting one of the main tourist attraction in the region. Furthermore, the region also has great conservation significance for small coastal cetaceans such as Franciscanas (*Pontoporia blainvillei*) and Guiana dolphins (*Sotalia guianensis*) (de Freitas Netto and Di Beneditto 2007, 2008, de Freitas Netto and Siciliano 2007).

In November 2015, the marine environment of Espírito Santo suffered drastic impacts from the greatest environmental disaster in Brazil’s history, the Mariana dam disaster. After the rupture of an iron ore tailings dam, approximately 60 million cubic meters of mining waste were washed into the Doce River at the Mariana municipality, Minas Gerais state. The toxic brown mud released by this disaster flowed through the Doce River until reaching the sea at Linhares, Espírito Santo. The toxic effects of the heavy metal-contaminated mudflow became a major concern for the marine fauna along the Espírito Santo coast (Frainer et al. 2016, Marta-Almeida et al. 2016, Miranda and Marques 2016, Gomes et al. 2018). While studies are still under way in order to evaluate the impacts of the Mariana dam disaster on the marine environment of Espírito Santo, this study provides a compilation of the data on cetaceans strandings in the 40 years preceding the disaster, providing a historical baseline for the region.

## Materials and methods

### Study area

The coastline of Espírito Santo state extends approximately 392 km from Riacho Doce stream (18.3475S, 39.6692W) to Itabapoana River (21.3067S, 40.9583W) (Figure 1A). The northern coast of Espírito Santo corresponds to the southern half of the Abrolhos Bank, a sediment-capped volcanic bank that encompasses the most important coralline reefs of the South Atlantic (Moura et al. 2013). Southeast from the Abrolhos Bank, the Vitoria-Trindade ridge is a series of volcanic seamounts that extends east, culminating in Trindade Island and Martim Vaz Archipelago approximately 1,200 km from the mainland (Fainstein and Summerhayes 1982). The coastal waters of Espírito Santo are dominated by tropical oligotrophic waters of the Brazilian current coming from the north, but are also under the influence of the seasonal coastal upwelling from the south (Schmid et al. 1995). The region combines coral reef ecosystems in the north and rocky reefs ecosystems in the south (transitioning at approximately 19°S) (Floeter et al. 2001). The highest primary biological productivity occurs at a 50 km-wide strip along the continental coastline, but significant biological productivity also occurs on the Abrolhos Bank and adjacent areas (Figure 1C) (NASA Earth Observatory 2019). The tropical monsoon climate is characterized by intensive and prolonged rains in summer and dry weather in winter, with east and northeast winds predominating in the summer and south and southeast winds occurring intermittently during winter (Nimer 1989). There are a number of river mouths along the coast of Espírito Santo, the most significant being the Doce River with an average flow ranging from 190 m^3^/s (September) to 650 m^3^/s (January) (Pinto et al. 2015). Human population density is highest on the southern half of Espírito Santo state, with a particularly high density on the coast of Vitória and Vila Velha municipalities (Figure 1C) (Centro Internacional de Agricultura Tropical et al. 2005); in contrast, Martim Vaz Archipelago is uninhabited and Trindade Island (Figure 1B) has a small military meteorological station with less than 40 people year-round.

### Data compilation

Data on strandings was derived from the published literature (including detailed data from articles previously published by the authors), publicly available reports, museum specimens and newspaper articles. Records of by-caught individuals that were brought ashore for necropsy were also included. Records of cetacean strandings were compiled from 01 January 1975 to 30 September 2015. The region is breeding grounds to *M. novaeangliae*, and this species has been the subject of long-term research efforts (Martins et al. 2013, Bezamat et al. 2015) and its strandings were not included in this report and will be analyzed separately.

It should be noted that beach monitoring effort was considerably irregular during the study period. Until 2010, with the exception of a brief period of systematic beach surveys in 1989 (Ramos et al. 1994, Siciliano 1995, Siciliano et al. 2002), the detection of strandings largely relied on the public reporting them to local NGOs and researchers. In October 2010, the Campos and Espírito Santo Basins Beach Monitoring Project (Projeto de Monitoramento de Praias das Bacias de Campos e Espírito Santo–PMP-BC/ES) was initiated as a requirement by federal environmental authorities for licensing of oil production in the region. Through PMP-BC/ES, the entire coastline of Espírito Santo was monitored by land on a daily basis, and any marine mammal strandings were recorded in a standardized manner.

For each stranding, the following data was compiled: species, date, sex (male, female, unknown sex), age group (calf, juvenile, adult, unknown age), and location. Geographic coordinates (decimal latitude and longitude, Datum WGS1984) were obtained for each stranding and were classified as: (a) “precise” when they were reported by the original source and indicated a location on the coastline of Espírito Santo, (b) “adjusted” when the coordinates provided by the original source were not sufficiently precise to indicate a location on the coastline, and had to be adjusted to represent the nearest location on the coastline, or (c) “approximate” when no coordinate was provided by the original source and an approximate location on the coastline was derived from a text description (e.g., name of the beach or city).

### Spatial and statistical analyses

Geographic coordinates were plotted and used to create kernel density heat maps using ArcGIS 10 (ESRI, Redlands, CA, USA). Although the Trindade and Martim Vaz islands are part of Vitória municipality, they were considered separately. The species discovery curve was obtained by plotting the cumulative number of species recorded as the number of strandings increased chronologically. For each stranding, the lunar cycle day (LCD) was calculated, in days, by subtracting the date of the preceding New Moon (obtained from United States Naval Observatory 2019) from the date of stranding. The lunar phase of each stranding was then classified as: (a) “Waxing” when 4 ≤ LCD ≤ 10, (b) “Full” when 11 ≤ LCD ≤ 17, (c) “Waning” when 18 ≤ LCD ≤ 24, and (d) “New” when LCD ≤ 3 or LCD ≥ 25. One-proportion tests were used to determine whether the proportion of males and females was different from an even distribution (excluding individuals of unknown sex) for the six most frequent species. Chi-Square tests were used to determine whether the number of strandings was heterogeneously distributed between the two most frequent species in relation to age groups, lunar phases and months. Significance level was 0.05 for all tests.

## Results

**Figure 2.**
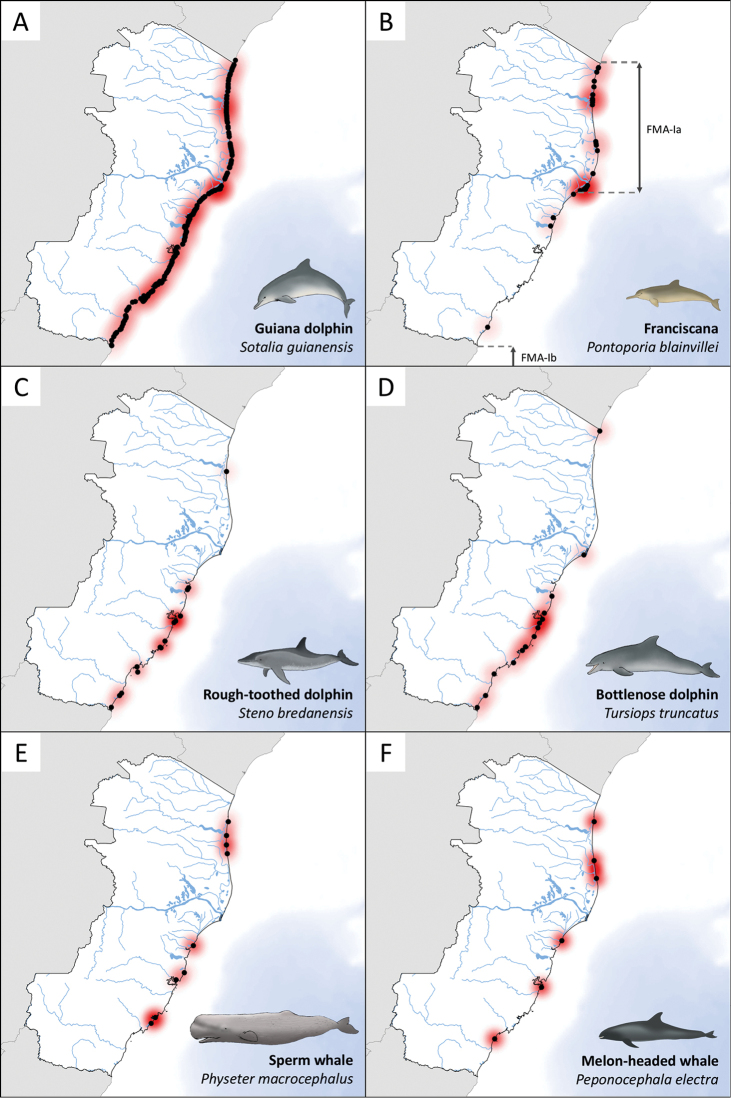
Geographic distribution of the six most frequently stranded cetacean species (excluding *Megaptera novaeangliae*) along the coast of Espírito Santo state, southeast Brazil, from January 1975 to September 2015. The limits of the Franciscana Management Areas (FMA) are shown in **B**.

**Figure 3.**
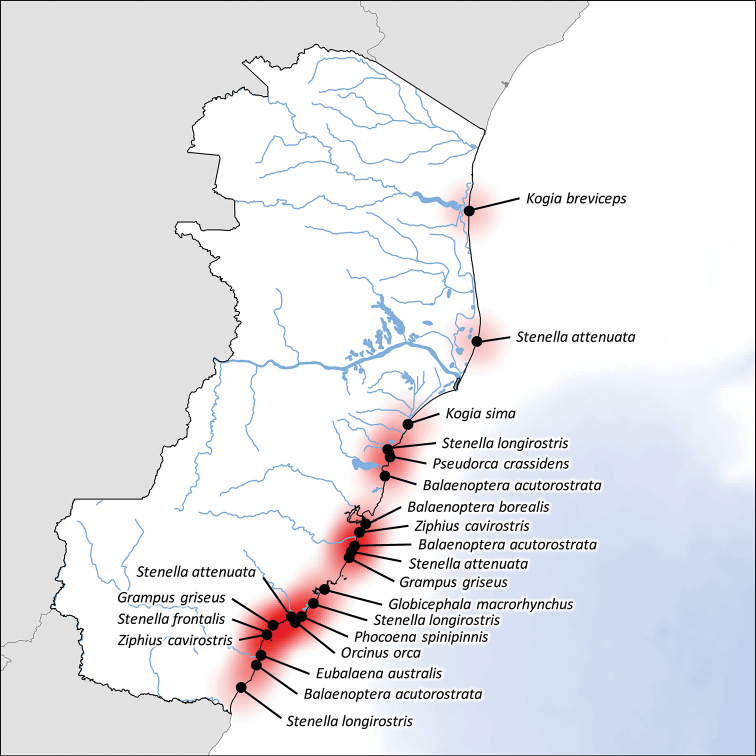
Geographic distribution of the least frequently stranded cetacean species along the coast of Espírito Santo state, southeast Brazil, from January 1975 to September 2015.

**Figure 4.**
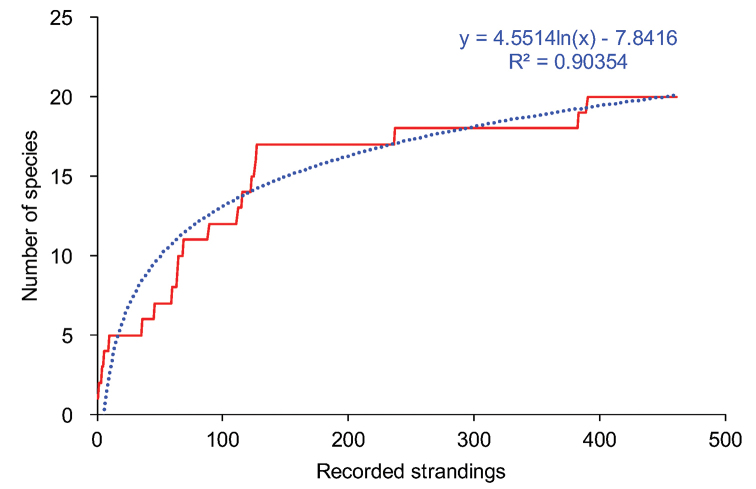
Species discovery curve for the number of cetacean species occurring in Espírito Santo waters based on stranding recordings.

**Figure 5.**
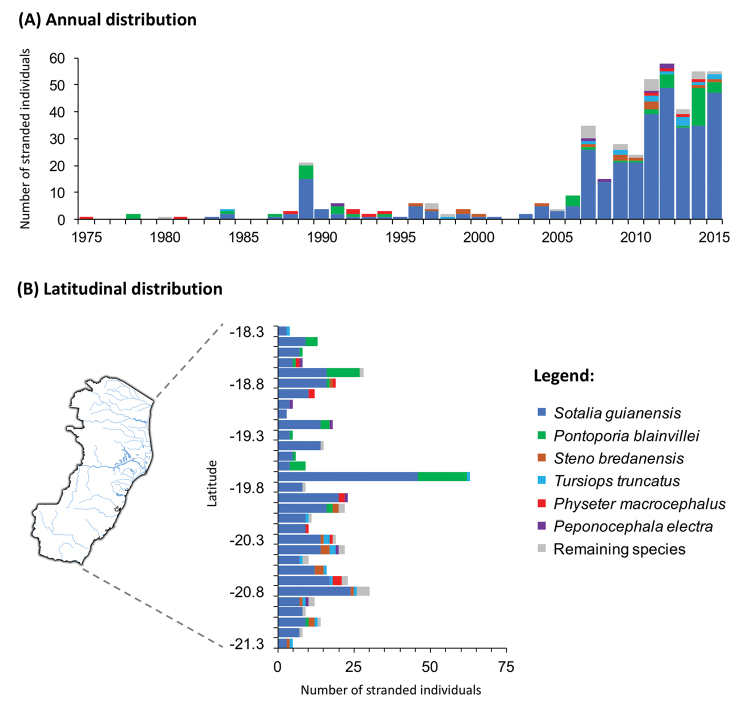
Annual and latitudinal distribution of recorded cetacean strandings (excluding *Megaptera novaeangliae*) along the coast of Espírito Santo state, southeast Brazil, from January 1975 to September 2015.

A total of 461 strandings was recorded, representing 20 cetacean species (Table 1, Suppl. material 1). Odontoceti corresponded to 456 individuals (98.9%) and 17 species (85%), and Mysticeti corresponded to 5 individuals (1.1%) and 3 species (15%). Six species had more than five recorded strandings: Guiana dolphin (*Sotalia guianensis*, 344 individuals), Franciscana (*P. blainvillei*, 47), rough-toothed dolphin (*Steno bredanensis*, 15), common bottlenose dolphin (*Tursiops truncatus*, 14), sperm whale (*Physeter macrocephalus*, 11), and melon-headed whale (*Peponocephala electra*, 6). The remaining 14 species comprised 24 individuals. The spatial distribution of the mainland strandings is shown in Figures 2, 3. The species discovery curve is shown in Figure 4. The annual and latitudinal distribution of strandings is summarized in Figure 5; it should be noted that Figure 5B does not include two strandings of Cuvier’s beaked whales (*Ziphius cavirostris*) recorded at Trindade Island. There were on average 0.14 recorded strandings per 100 km per month until September 2010 (before PMP-BC/ES) and 1.18 recorded strandings per 100 km per month since October 2010 (during PMP-BC/ES).

**Table 1.** Recorded cetacean strandings (excluding *Megaptera novaeangliae*) along the coast of Espírito Santo state, southeast Brazil, from January 1975 to September 2015. The number of stranded individuals is represented as “Male : Female : Unknown sex”.

Family	Species	Common name	Calf	Juvenile	Adult	Unknown age	Total
Balaenidae	*Eubalaena australis*	Southern right whale	–	–	–	0:0:1	0:0:1
Balaenopteridae	*Balaenoptera acutorostrata*	Common Minke whale	1:0:0	0:1:0	–	1:0:0	2:1:0
	*Balaenoptera borealis*	Sei whale	–	–	–	0:0:1	0:0:1
Delphinidae	*Globicephala macrorhynchus*	Short-finned pilot whale	–	1:0:0	–	–	1:0:0
	*Grampus griseus*	Risso’s dolphin	1:0:0	–	1:0:0	–	2:0:0
	*Orcinus orca*	Orca	–	0:1:0	–	–	0:1:0
	*Peponocephala electra*	Melon-headed whale	0:1:0	0:0:1	2:1:0	0:0:1	2:2:2
	*Pseudorca crassidens*	False killer whale	–	–	–	0:1:0	0:1:0
	*Sotalia guianensis*	Guiana dolphin	5:3:7	33:19:37	54:24:66	10:1:85	102:47:195
	*Stenella attenuata*	Pantropical spotted dolphin	1:0:0	1:0:0	–	0:0:1	2:0:1
	*Stenella frontalis*	Atlantic spotted dolphin	–	–	0:1:0	–	0:1:0
	*Stenella longirostris*	Spinner dolphin	–	0:1:0	0:1:0	0:0:1	0:2:1
	*Steno bredanensis*	Rough-toothed dolphin	0:0:2	2:0:0	3:4:1	1:0:2	6:4:5
	*Tursiops truncatus*	Bottlenose dolphin	–	1:0:0	7:1:1	0:0:4	8:1:5
Kogiidae	*Kogia breviceps*	Pygmy sperm whale	–	–	–	0:0:1	0:0:1
	*Kogia sima*	Dwarf sperm whale	–	–	–	0:0:1	0:0:1
Phocoenidae	*Phocoena spinipinnis*	Burmeister’s porpoise	–	–	1:0:0	–	1:0:0
Physeteridae	*Physeter macrocephalus*	Sperm whale	1:0:0	0:1:0	1:0:0	1:0:7	3:1:7
Pontoporiidae	*Pontoporia blainvillei*	Franciscana	2:2:4	1:0:2	0:2:13	0:1:20	3:5:39
Ziphiidae	*Ziphius cavirostris*	Cuvier’s beaked whale	–	–	0:3:1	–	0:3:1
**Total**			11:6:13	39:23:40	69:37:82	13:3:125	132:69:260

The sex ratio was biased towards males in *S. guianensis* (*Z* = 4.516, *p* < 0.001; 95% CI of the proportion of males = 60.4–75.9%) and *T. truncatus* (*Z* = 2.334, *p* = 0.02; 95% CI = 51.8–99.7%). For the remaining species, sex ratio was not significantly different from an even distribution (all *p* > 0.30). The age distribution was different between *S. guianensis* and *P. blainvillei* (c^2^ = 21.288, *df* = 2, *p* < 0.001), with adults being the most frequent category in both species (respectively, 58% and 67%) but calves being more frequent in *S. guianensis* (31%) than in *P. blainvillei* (17%).

**Figure 6.**
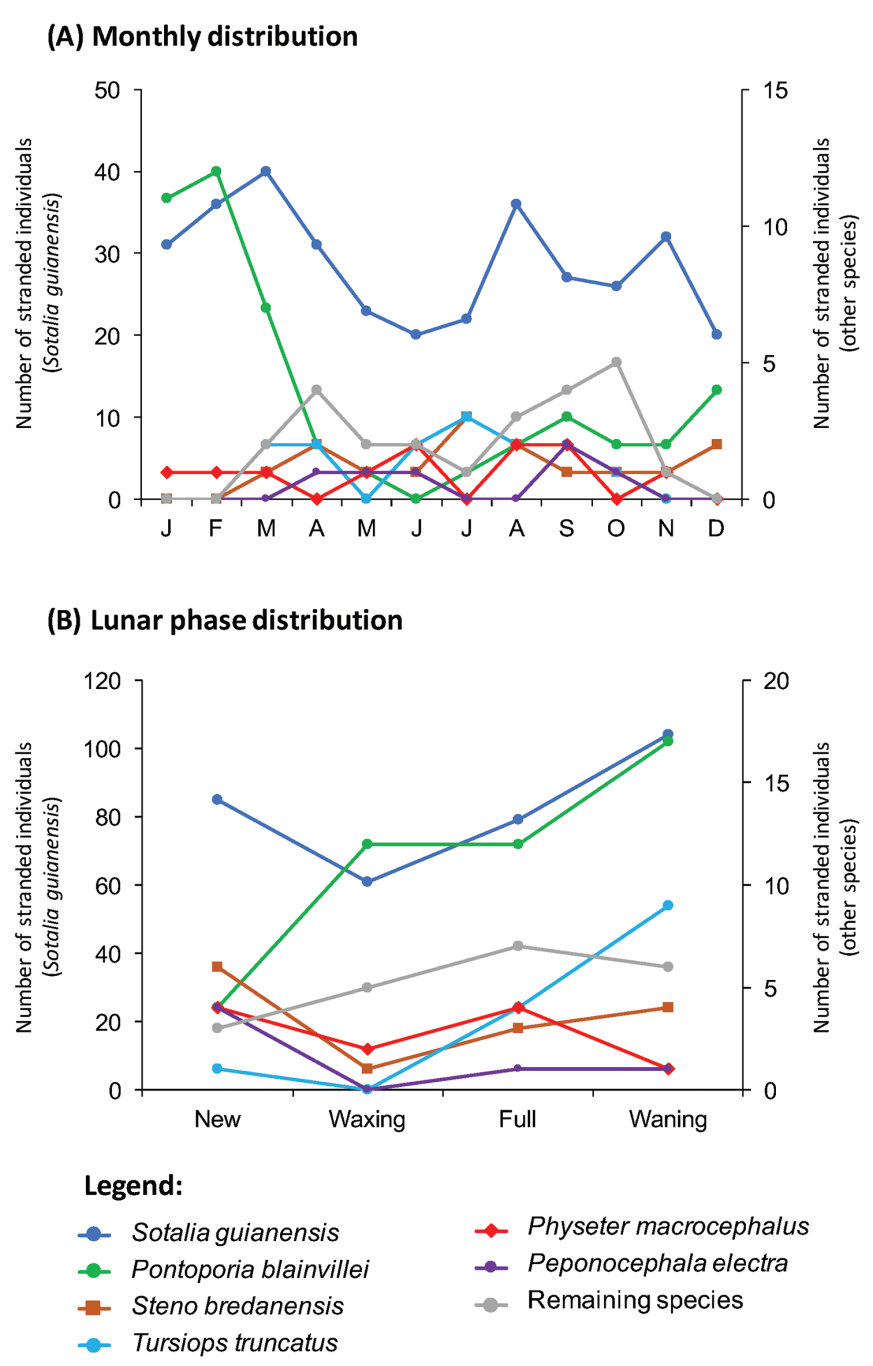
Monthly and lunar phase distribution of recorded cetacean strandings (excluding *Megaptera novaeangliae*) along the coast of Espírito Santo state, southeast Brazil, from January 1975 to September 2015.

The monthly distribution of strandings of *S. guianensis* and *P. blainvillei* was significantly different (c^2^ = 26.596, *df* = 11, *p* = 0.005) with *S. guianensis* strandings occurring year-round with peaks in March, August and November, whereas those of *P. blainvillei* were predominantly concentrated from January to March (Figure 6A). The strandings were similarly distributed in *S. guianensis* and *P. blainvillei* (c^2^ = 6.697, *df* = 3, *p* = 0.082), with both species (along with *T. truncatus*) presenting a higher number of strandings during waning moon (Figure 6B).

## Discussion

Twenty species were recorded in this study (21 spp. if humpback whales are included), representing nearly half of Brazil’s known diversity of cetaceans (45 spp.) (Paglia et al. 2012). The cetacean fauna of Espírito Santo is largely composed by small and medium-sized tropical coastal and oceanic species, similarly to that of the Northeastern coast of Brazil (Meirelles et al. 2009, Batista et al. 2012), however with fewer Caribbean species and species related to upwelling fronts such as pantropical and Atlantic spotted dolphins (*Stenella attenuata* and *Stenella frontalis*, respectively) and Bryde’s whales (*Balaenoptera brydei*) (Costa et al. 2017). In certain respects, the cetacean community of Espírito Santo arguably bears similarity to that other pantropical oligotrophic regions such as the Western Tropical Indian Ocean, the Eastern Tropical Pacific and the Gulf of Mexico (Ballance and Pitman 1998). The species discovery curve suggests that the discovery rate has slowed down but a plateau has not yet been reached, and logarithmic regression suggests an additional three species would have been recorded if the number of recorded strandings had been doubled. The vast majority (99%) of the individuals in this study were odontocetes, however this is a biased proportion because we did not include humpback whales in the dataset (their strandings will be analyzed separately).

The average number of recorded strandings increased by 750% after the start of a daily beach survey program (PMP-BC/ES), illustrating how the occurrence of strandings can be greatly underestimated in the absence of such survey efforts. During the period when daily beach surveys were conducted (October 2010 to September 2015), there was a relatively low rate of 1.2 recorded strandings per 100 km per month. This is not an unexpected result, considering the oligotrophic tropical waters of the region (Schmid et al. 1995). In comparison, a similar beach survey program at the Paraná and Santa Catarina states, southern Brazil, has recorded an average of 6.6 strandings per 100 km per month (data from 01/01/2016-31/12/2018, excluding humpback whales; Projeto de Monitoramento de Praias 2019).

Six species were most frequent and represented nearly 95% of all strandings: *S. guianensis* (74.6%), *P. blainvillei* (10.2%), *S. bredanensis* (3.3%), *T. truncatus* (3.0%), *P. macrocephalus* (2.4%) and *P. electra* (1.3%). The fact that the two most frequently recorded species (*S. guianensis* and *P. blainvillei*) are coastal-dwelling suggests that the stranding probability might be influenced by the natural distribution of these species, and therefore stranding data might systematically underrepresent the abundance of pelagic species in the continental waters of Espírito Santo.

Interestingly, strandings were unevenly distributed with regards to the lunar phase. Previous studies have obtained contradictory results in this respect, showing that strandings may be more frequent during full or new moon in New Zealand and Canada (Cordes 1982, Lad and Brabyn 1993, Wright 2005) and during waxing moon in Great Britain (Wright 2005). In this study, strandings of Guiana dolphins, Franciscanas and Common bottlenose dolphins, were most frequent in waning moon, whereas no evident pattern was noted for the remaining species. While this ecological dynamic is not yet understood, potential factors that may drive uneven lunar distribution in cetacean strandings include changes in tidal magnitude (Cordes 1982, Lad and Brabyn 1993, Wright 2005), behavioral responses to geomagnetism (Kirschvink et al. 1986, Nishimura and Fukushima 2009) and planktonic growth and migration cycles (Hernandez-Leon et al. 2002, Last et al. 2016).

### Guiana dolphin, *Sotalia guianensis* (van Bénéden, 1864)

Guiana dolphins were frequently recorded throughout the Espírito Santo coast, with hotspots of occurrence in estuaries. The Doce River estuary was a particularly significant hotspot for strandings of this species, in agreement with previous studies that suggested this is an important habitat for the Guiana dolphin (Pinheiro 2014).

Previous studies found that most Guiana dolphins stranded in Brazil are immature males (Lima et al. 2017), which is consistent with the sex ratio of 68% males recorded in this study. The species is known to be frequently bycaught in gillnets in Espírito Santo (Barros et al. 1997, de Freitas Netto and Barbosa 2003, de Freitas Netto and Di Beneditto 2008), especially before reaching sexual maturity, with 80% of bycaught individuals corresponding to male individuals younger than 6 years (Rosas et al. 2003, Di Beneditto and Ramos 2004). The finding that stranded individuals were also predominantly young males (at least for the subset of 149 individuals for which sex was determined, bearing in mind that sex was not determined for 57% of Guiana dolphin strandings in this study) suggests that a substantial proportion of the strandings recorded in this study might correspond to individuals that were bycaught in gillnets and then washed ashore. The spatial hotspots and temporal distribution of Guiana dolphin strandings could therefore be partly related to an uneven distribution in gillnet fishing effort rather than being exclusively explained by the species’ ecology (de Freitas Netto and Di Beneditto 2007, Prado et al. 2016). In this context, the large number of strandings at the Doce River estuary might be interpreted as the result of the overlap of the high density of Guiana dolphins with a particularly intense local gillnet fishing effort (Pinheiro 2014). Furthermore, bycatch could also help explaining the February-March peak in strandings, potentially reflecting increased fishing efforts in anticipation to the Easter holidays.

The Guiana dolphin is a regionally abundant species that is highly sensitive to environmental impacts such as fisheries bycatch (Rosas et al. 2003, Di Beneditto and Ramos 2004), human disturbance (Filla et al. 2009), chemical pollution (Lailson-Brito et al. 2010, Moura et al. 2014) and pathogen outbreaks (Gonzales-Viera et al. 2013, Groch et al. 2018). These characteristics, in addition to being classified as Near Threatened (Secchi et al. 2019), render the Guiana dolphin a prime candidate to serve as an ecosystem sentinel for the coastal waters of eastern and southeastern Brazil (Moura et al. 2014).

### Franciscana, *Pontoporia blainvillei* Gervais & d’Orbigny, 1844

The population of Franciscanas is split into four Franciscana Management Areas (FMA) based on its ecogeography, morphology and genetics (Secchi et al. 2003, Cunha et al. 2014). The study area corresponds to FMA-Ia, which extends from the northern limit of the Espírito Santo state (18.3S) to the Doce River estuary (19.7S) (Figure 2B). The species’ distribution has a hiatus of approximately 220 km from Doce River to the southern limit of Espírito Santo (21.3S), where FMA-Ib begins (Cunha et al. 2014, Danilewicz 2018). Our finding that there were only three recorded strandings within the hiatus in 40 years is in agreement with genetic studies that indicated limited gene flow between FMA-Ia and FMA-Ib (Cunha et al. 2014).

Most strandings occurred near the mouth of large rivers, especially of the Doce and São Mateus rivers, which is consistent with the species’ reliance on turbid waters to feed (Siciliano et al. 2002). Doce River is recognized as a particularly important foraging area within FMA-Ia, thanks to its high productivity and turbidity (Siciliano et al. 2002). The strandings of Franciscana also showed marked seasonality, with 64% of the records occurring from January to March. This pattern matches the summer increases in rainfall and river flow (Pinto et al. 2015), which might encourage the species to come closer to the shore to take advantage from the increased biological productivity. It is also worth noting that although the presence of lesions suggestive of bycatch was not systematically recorded, at least 14 individuals (32%) had clear indications of interaction with fisheries such as net marks, rostral fractures or attached ropes/nets.

The Franciscana is currently classified as Vulnerable (Secchi et al. 2019), and FMA-Ia is the smallest and least-studied population of Franciscanas, with a remarkably low genetic diversity and gene flow to/from other populations (Secchi et al. 2003, Cunha et al. 2014), and an estimated population of 653 individuals (Danilewicz 2018). With an average of 0.43 strandings per month in recent years (since daily beach surveys were implemented), it may be estimated that the annual number of recorded strandings corresponds to ca. 0.8% of the FMA-Ia population. This is likely an underestimation of the true mortality rate, as in other studies the number of stranded cetacean carcasses has been found to represent between 0% and 6.2% of the total mortality, depending on the species (Williams et al. 2011). If the 6.2% upper estimate is used, this would imply that the true mortality might be 16.1 times higher than the number of recorded strandings, i.e., an annual mortality of ca. 12.9% of the FMA-Ia population. It is therefore clear that the survival of the Franciscana population at Espírito Santo requires urgent efforts to mitigate the human impacts, especially at the estuaries on the northern coast of the state (Doce, São Mateus, Mariricu and Itaúnas rivers).

### Rough-toothed dolphin, *Steno bredanensis* G. Cuvier in Lesson, 1828

With the exception of one record at São Mateus, the strandings of rough-toothed dolphins were limited to the southern portion of Espírito Santo, where the continental platform is narrower. This may be related to the dietary habits of this species, which feeds predominantly on mesopelagic fish (depth 200–1,000 m) (Berta 2015), and therefore likely prefers to forage in deeper waters than those of the Abrolhos Bank. The recorded strandings of rough-toothed dolphins also appear to concentrate near the larger cities of the southern coast (Vitória/Vila Velha and Guarapari/Anchieta), which might reflect a greater probability of opportunistic detection prior to the daily beach survey program. It is worth noting that one of these strandings involved an adult male that starved due to large quantities of plastic bags in its gastrointestinal tract (Bhering et al. 2010), illustrating the impacts that marine pollution can have on this species (Meirelles and Barros 2007). It is also interesting to note that several rough-toothed dolphin strandings occurred in pairs, with two individuals stranding in the same general area within a few months (e.g., records A370 and A371, A375 and A376, A378 and A379 in Suppl. material 1). It is unclear whether this is a coincidence or if instead it represents a group/familiar dynamic (e.g., mother-calf pairs), and further studies are warranted to evaluate this possibility.

### Common bottlenose dolphin, *Tursiops truncatus* (Montagu, 1821)

With the exception of two records (Conceição da Barra and Linhares municipalities), the strandings of common bottlenose dolphins were limited to the southern portion of Espírito Santo. However, unlike the rough-toothed dolphin, common bottlenose dolphins have a flexible diet that includes prey from coastal waters (Wells and Scott 2009), and therefore it would be reasonable to expect this species would be common in the Abrolhos Bank. A possible explanation is that the species is concentrating at the Vitória eddy (Schmid et al. 1995). Because the Greater Vitória metropolitan area was the main hotspot for common bottlenose dolphin strandings, another potential explanation would be that these animals are attracted by the fisheries targeting small pelagic fishes that operate from that area and with which this species is known to interact (Zappes et al. 2011). It is worth noting that although the Itaipava fishing fleet is the largest in the state, it focuses primarily on longline fishing of dolphinfish and tuna (Bugoni et al. 2008), which would not be attractive for common bottlenose dolphins.

### Sperm whale, *Physeter macrocephalus* Linnaeus, 1758

Sperm whale strandings were diffusely distributed along the Espírito Santo coast, without well-defined hotspots. At-sea observations suggest that sperm whales tend to concentrate along the central coast of Bahia, especially at the Camamu-Almada Basin, and occur at much lower densities in Espírito Santo (Batista et al. 2012). The number of strandings of this species in this study was relatively high compared to similar surveys conducted in other portions of the Brazilian coastline (Prado et al. 2016, Vianna et al. 2016, Costa et al. 2017), which suggests this species probably forages along the Vitória-Trindade ridge and occasionally approaches the Espírito Santo coast. It is worth noting that previous studies suggested that the species might be most abundant in Espírito Santo waters in winter and spring, from July to November (Batista et al. 2012), but this was not evident in the strandings recorded in this study.

### Melon-headed whale, *Peponocephala electra* (Gray, 1846)

Melon-headed whales are relatively common on the coast of northeast Brazil, including Bahia, where a mass stranding event involving 240 individuals was recorded on 1987 (Lodi et al. 1990). Strandings were diffusely distributed along the Espírito Santo coast, without well-defined hotspots. The number of strandings of this species in this study was relatively high compared to similar surveys conducted in other portions of the Brazilian coastline (Prado et al. 2016, Vianna et al. 2016, Costa et al. 2017), which is consistent with at-sea surveys showing that melon-headed whales are occasionally seen along the coast of Espírito Santo (Wedekin et al. 2009, 2014). It is worth noting that there was one instance where a live individual stranded in the state, but it later died and its death was attributed to plastic ingestion (Costa et al. 2012), illustrating the impacts of plastic pollution can have on this species.

### Other species

The remaining 14 species comprised 24 individuals, predominantly corresponding to small species with tropical oceanic distribution. Most of these records were at the southern portion of Espírito Santo, which suggests that the narrower continental platform may have increased the probability of stranding.

The only baleen whales recorded in this study were common Minke whale (*Balaenoptera acutorostrata*), Sei whale (*Balaenoptera borealis*) and southern right whale (*Eubalaena australis*). These species are known to occasionally occur in the region during their winter migration in the Southwest Atlantic (Zerbini et al. 1997, Berta 2015). Based on their predicted distribution, the following mysticetes that would be expected to occur in the study area (besides humpback whales) but were not recorded in this study are: Antarctic Minke whales (*Balaenoptera bonaerensis*), Bryde’s whales, and Blue whales (*Balaenoptera musculus*); the southern coast of Espírito Santo is also on the distribution limit for the Fin whale (*Balaenoptera physalus*) (Berta 2015, International Union for Conservation of Nature and Natural Resources 2019).

With the exception of the Burmeister’s porpoise (*Phocoena spinipinnis*), the remaining records of odontocetes are within the known distribution of each species (Berta 2015, International Union for Conservation of Nature and Natural Resources 2019). Burmeister’s porpoises are small odontocetes that inhabit the coastal waters of South America (Berta 2015). Although the species’ distribution extends as far north as 5°46'S on the Pacific coast (northern Peru), its distribution on the Atlantic coast is generally considered to be restricted south of 27°55'S (Santa Catarina state, Brazil) (International Union for Conservation of Nature and Natural Resources 2019). This species is rare in southern Brazil, with only one record in Santa Catarina and six records in Rio Grande do Sul despite over 30 years of systematic beach surveys (Prado et al. 2016, Vianna et al. 2016). Therefore, the Burmeister’s porpoise recorded in Espírito Santo, more than 1,120 km from the species’ northern Atlantic distribution limit, clearly represents an extra-limital record. This could be related to a straggling individual or perhaps a carcass brought by fishing vessels. The later hypothesis is strengthened by the fact that the specimen stranded at Anchieta municipality, one of the ports of the Itaipava fishing fleet which is known to operate along the South Brazil Bight (continental waters from Rio Grande do Sul to São Paulo) (Bugoni et al. 2008).

Based on their distribution range, the following odontocetes would also have been expected to occur in the study area but were not recorded in this study: long-beaked common dolphin (*Delphinus capensis*), short-beaked common dolphin (*Delphinus delphis*), pygmy killer whale (*Feresa attenuata*), Fraser’s dolphin (*Lagenodelphis hosei*), Blainville’s beaked whale (*Mesoplodon densirostris*), Clymene dolphin (*Stenella clymene*), and striped dolphin (*Stenella coeruleoalba*) (Berta 2015, International Union for Conservation of Nature and Natural Resources 2019).

### Implications for conservation

Our results suggest that the Doce River estuary may be a particularly significant area for cetaceans in Espírito Santo, especially Guiana dolphins and Franciscanas. For this reason, the impacts that the Mariana dam disaster may have had on these species, both of which were already threatened with extinction, are acutely concerning. The mudflow resulting from this incident had a high concentration of heavy metals (Frainer et al. 2016, Marta-Almeida et al. 2016, Miranda and Marques 2016, Gomes et al. 2018), and cetaceans are particularly at risk of intoxication due to biomagnification along the food chain (Fossi and Panti 2018). Furthermore, the extent of the mudflow led to substantial impacts to the marine environment which likely also affected prey availability for these species (Frainer et al. 2016, Miranda and Marques 2016). Environmental impact studies to evaluate the consequences of this disaster on the marine fauna of Espírito Santo are underway, and our results will serve as a baseline for comparison for post-2015 stranding data (e.g., number and species of strandings, seasonal distribution, etc.).

Four threatened species were recorded in this study: Guiana dolphin (Vulnerable), Franciscana (Vulnerable), sperm whale (Vulnerable), and Sei whale (Endangered) (International Union for Conservation of Nature and Natural Resources 2019). It is remarkable that three of these species (Guiana dolphin, Franciscana, and sperm whale) were amongst the most frequently recorded in this study. Considering their differences in habitat use and body size, it is clear that these factors alone could not explain their particularly high frequency. Instead, our data suggest that these species are either particularly abundant or experience high mortality in the study area. In particular, the Franciscana presents a critical conservation challenge, as its small population in northern Espírito Santo is known to be genetically distinct and geographically isolated (Secchi et al. 2003, Cunha et al. 2014, Danilewicz 2018), and our results suggest that this population already experienced high annual mortality prior to the Mariana dam disaster.

Although the seamounts along the Vitória-Trindade ridge likely provide attractive habitat for marine fauna (Floeter et al. 2007, Pinheiro et al. 2015a, b), including cetaceans (Wedekin et al. 2014), only two strandings were recorded at Trindade Island, both of Cuvier’s beaked whales. Such a small number of recorded strandings is likely due to a combination of the relatively small size of the island (6.1 × 2.2 km), the small human population (< 40 inhabitants) and the lack of systematic beach surveys (which would be extremely difficult due to the steep terrain). The lack of recorded strandings therefore should not be interpreted as an indication that this region is not a significant habitat for cetaceans. The impacts that the mudflow from the Mariana dam disaster may have had on cetacean communities along the Vitória-Trindade ridge therefore also merits careful consideration.

Lastly, it is worth highlighting that strandings of sperm whales and melon-headed whales occurred with an unexpectedly high frequency in comparison to other similar surveys conducted in portions of the Brazilian coastline (Meirelles et al. 2009, Prado et al. 2016, Vianna et al. 2016, Costa et al. 2017), which suggests that these deep-oceanic species might aggregate off the southern coast of Espírito Santo. Additional studies employing aerial and boat transects, telemetry and acoustic surveys are therefore warranted to investigate this hypothesis and determine important areas for cetaceans in the region.

## Supplementary materials

Supplementary material 1 Historical records of cetacean strandings along the coast of Espírito Santo state, southeast Brazil, 1975–2015 Data type occurrences Explanation note List of the historical records of cetacean strandings along the coast of Espírito Santo state, southeast Brazil, including information on the date, location, coordinates, species, age group, sex, musem accession code, field observations and reference/source. https://binary.pensoft.net/file/431571This dataset is made available under the Open Database License (http://opendatacommons.org/licenses/odbl/1.0/). The Open Database License (ODbL) is a license agreement intended to allow users to freely share, modify, and use this Dataset while maintaining this same freedom for others, provided that the original source and author(s) are credited.Luis Felipe S. P. Mayorga, Ralph E. T. Vanstreels, Renata C. C. Bhering, Natália Mamede, Luiz M. B. Costa, Flavia C. F. Pinheiro, Luciano W. D. Reis, Alessandro Trazzi, Wilson Luiz Chevitarese Meirelles, Alan Marques Ribeiro, Salvatore Siciliano
